# Pericardial Effusion Secondary to Amoebic Liver Abscess: A Rare Complication

**DOI:** 10.7759/cureus.11759

**Published:** 2020-11-28

**Authors:** Mohammad Raza, Sohail Kumar, Deedar Nanjiani, Azhar Hussain, Saad Azizullah

**Affiliations:** 1 Pediatrics, The Indus Hospital, Karachi, PAK; 2 Pediatrics, Dow Medical College, Dr. Ruth K. M. Pfau Civil Hospital, Karachi, PAK; 3 Internal Medicine, Dow Medical College, Dr. Ruth K. M. Pfau Civil Hospital, Karachi, PAK; 4 Healthcare Administration, Franklin University, Columbus, USA; 5 Medicine, Xavier University School of Medicine, Oranjestad, ABW

**Keywords:** amoebic liver abscess, pericardial effusion, entamoeba histolytica, pericardial amoebiasis, complication

## Abstract

Amoebiasis is a common infection widely prevalent in tropical countries with low income and poor sanitation. The clinical picture is usually nonspecific; however, invasion of the liver by *Entamoeba histolytica* could lead to an amoebic liver abscess (ALA). It is relatively uncommon in women and children. Though rare, extension of ALA into the lungs, pleural cavity, and pericardium may prove fatal. Pericardial amoebiasis is a rare complication which, if not treated early, could result in cardiac tamponade and subsequent death. The standard management option is eradication with metronidazole along with the drainage of fluid from the liver abscess and pericardial effusion. Herein, we present a case of a seven-year-old male child with ALA, who developed signs and symptoms suggesting pericardial effusion within a few days of hospital admission. Early diagnosis of pericardial complication and successful management of abscess resolved the pericardial effusion.

## Introduction

Amoebiasis is an infection caused by *Entamoeba histolytica*, a potential parasitic protozoan transmitted through the fecal-oral route. It commonly occurs by the ingestion of cysts present in contaminated food and water. Such parasitic infections, previously limited to tropical areas, have now become a global problem, especially in countries with inadequate sanitary systems. Although amoebiasis typically presents with symptoms such as abdominal pain and bloody diarrhea, migration of the trophozoite from the intestine to the liver can also occur, resulting in an amoebic liver abscess (ALA). ALA is the most common extra-intestinal manifestation of amoebiasis. It is uncommon in children and women. Poor hygiene, malnutrition, immunosuppression, poor socioeconomic status, and recent travel to an endemic area are potential risk factors [1,2].

In rare cases, the infection may manifest as peritonitis, pleuropulmonary, cardiac, or brain disease either directly from rupture of liver abscess or through dissemination into the blood. Rupture of an amoebic liver abscess into the pericardial cavity is a rare and highly dangerous complication. It occurs in less than 2% of cases, in which the ALA occurs in the left lobe of the liver [3,4]. Pericardial amoebiasis presents with symptoms such as fever, chest pain, and progressive dyspnea, which may eventually progress into a potentially life-threatening cardiac tamponade [3,4]. The diagnosis is made based on clinical presentation with relevant epidemiology along with radiographic and serologic findings. Early management is necessary to lessen the risks of complications and reduce the chances of mortality.

Herein, we report a case of pericardial effusion, occurring as a rare complication of amoebic liver abscess in a seven-year-old male child. Effusion was diagnosed at an early stage with the help of echocardiography and was treated successfully with conservative measures. 

## Case presentation

A seven-year-old male child presented to The Indus Hospital, Karachi, with the chief complaints of fever and abdominal pain for 15 days and diarrhea for three days. The frequency of diarrhea was two to three episodes per day. It was foul-smelling and did not contain any blood or mucus. Fever was high-grade and intermittent, with associated chills and sweating. The pain was concentrated in the epigastric and right hypochondriac regions. It was dull and continuous in nature and accompanied by nausea, vomiting, and decreased appetite. The patient took antipyretics and oral antibiotics prior to hospital admission, but his symptoms showed no improvement. The patient was the third product of a non-consanguineous marriage, and his birth history was unremarkable. He had been fully vaccinated, according to the Extended Program for Immunization (EPI). He achieved all of his developmental milestones at an appropriate age. His past medical history revealed that he was hospitalized at 2.5 years of age due to acute gastroenteritis.

On physical examination, the child was lethargic, dehydrated, febrile (39 °C), and slightly tachypneic (32 breaths/minute). The heart rate (110 beats/minute) and blood pressure (110/70mmHg) were normal. There was no sign of cyanosis, jaundice, pedal edema, or lymphadenopathy. An abdominal examination revealed a soft and tender abdomen; the liver was enlarged and palpable 4 cm below the costal margin. The rest of the systemic examinations were unremarkable. 

Routine laboratory investigations at the time of admission revealed a low hemoglobin count (due to iron deficiency) but a high total leukocyte count (predominantly neutrophils). Sodium and bicarbonate were mildly reduced. Chloride, potassium, albumin, and liver function tests (LFTs) were all within normal limits. C-reactive protein (CRP) and erythrocyte sedimentation rate (ESR) were markedly elevated. The laboratory investigations of the patient are summarized in Table 1. 

**Table 1:** Laboratory investigations of the patient Hb, hemoglobin; RBC, red blood cell; Hct, hematocrit; MCV, mean corpuscular volume; MCH, mean corpuscular hemoglobin; MCHC, mean corpuscular hemoglobin concentration; TLC, total leukocyte count; RDW-CV, red blood cell distribution width; CRP, c-reactive protein; ESR, erythrocyte sedimentation rate; CK-MB, creatine kinase myocardial band.

Laboratory Investigation	Patient’s Result	Reference Range
Hb	7.6	13.0-17.0 g/dl
RBC Count	3.17	4.5-5.5 million/mcL
Hct	24.2	40-50%
MCV	76.4	80-100 fL
MCH	24	27-32 pg
MCHC	31.5	31.5-34.5 g/dL
TLC	15100	4,000-10,000/μL
Neutrophils	71.5	40-75%
Lymphocytes	15.3	20-45%
Monocytes	10.9	02-10%
Eosinophils	1.6	01-06%
Basophils	0.7	00-01%
Platelets	502,000	150,000-450,000/μL
RDW-CV	23.7	11.5-14.5%
Sodium	133.23	136-145 mEq/L
Bicarbonate	21	22-28 mEq/L
Chloride	101.23	95-105 mEq/L
Potassium	3.50	3.5-5.0 mEq/L
CRP	50.7	<3 g/dL
ESR	66	0-15 mm/h
Ferritin	193.89	12-300 ng/mL
Calcium	8.18	8.5-10.5 mg/dl
Iron	22	10.74-30.43 mmol/L
Magnesium	1.89	1.7-2.2 mg/dL
Phosphorus	5.10	2.5-4.5 mg/dL
Albumin	2.80	3.5-5.5 g/dL
Creatinine	0.42	0.6-1.2 mg/dL
CK-MB	13.00	5-25 IU/L
Vitamin D	7.8	20-50 ng/mL
Vitamin B12	334	200-900 ng/mL

**Figure 1:**
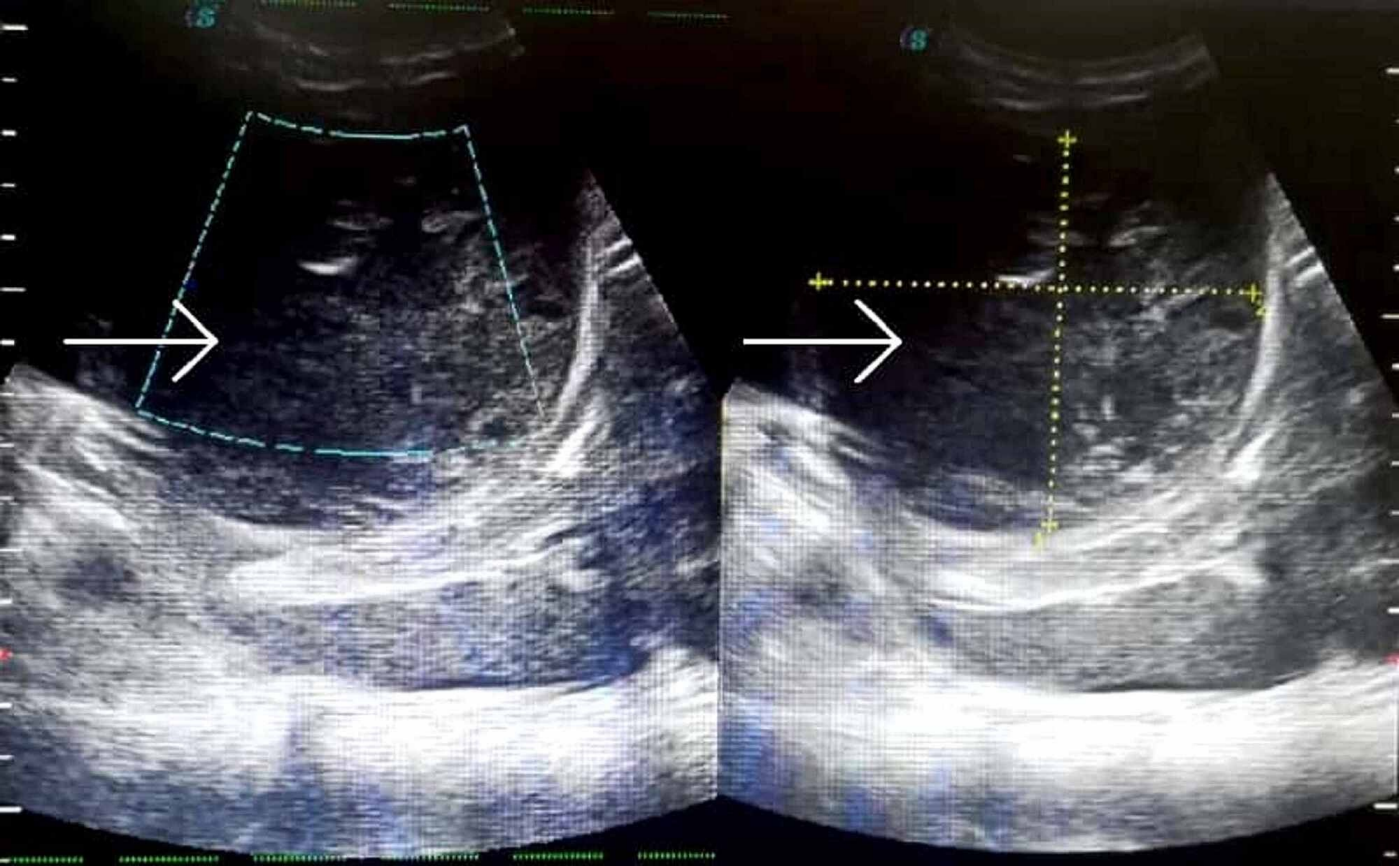
Abdominal ultrasound showing a large heterogeneous lesion, consistent with liver abscess, in the left lobe of the liver

Ultrasound (US) of the abdomen was performed, which revealed a large heterogeneous lesion, consistent with liver abscess, located predominantly in the left lobe of the liver measuring 7.7 x 8.8 cm in anteroposterior and transverse dimensions, as shown in Figure 1. The patient was immediately started on an empirical anti-amoebic therapy with metronidazole and ceftriaxone after sending blood culture; meanwhile, we ordered serology and stool samples for *E. histolytica*. Immediate drainage of the abscess was planned and performed under US guidance, followed by placement of a pigtail catheter to drain any residual collection. We ruled out pyogenic liver abscesses based on negative pus and blood culture reports. Eventually, the stool sample containing cysts of *E. histolytica* and positive enzyme-linked immunosorbent assay (ELISA) test led to the corroborative diagnosis of ALA. 

**Figure 2:**
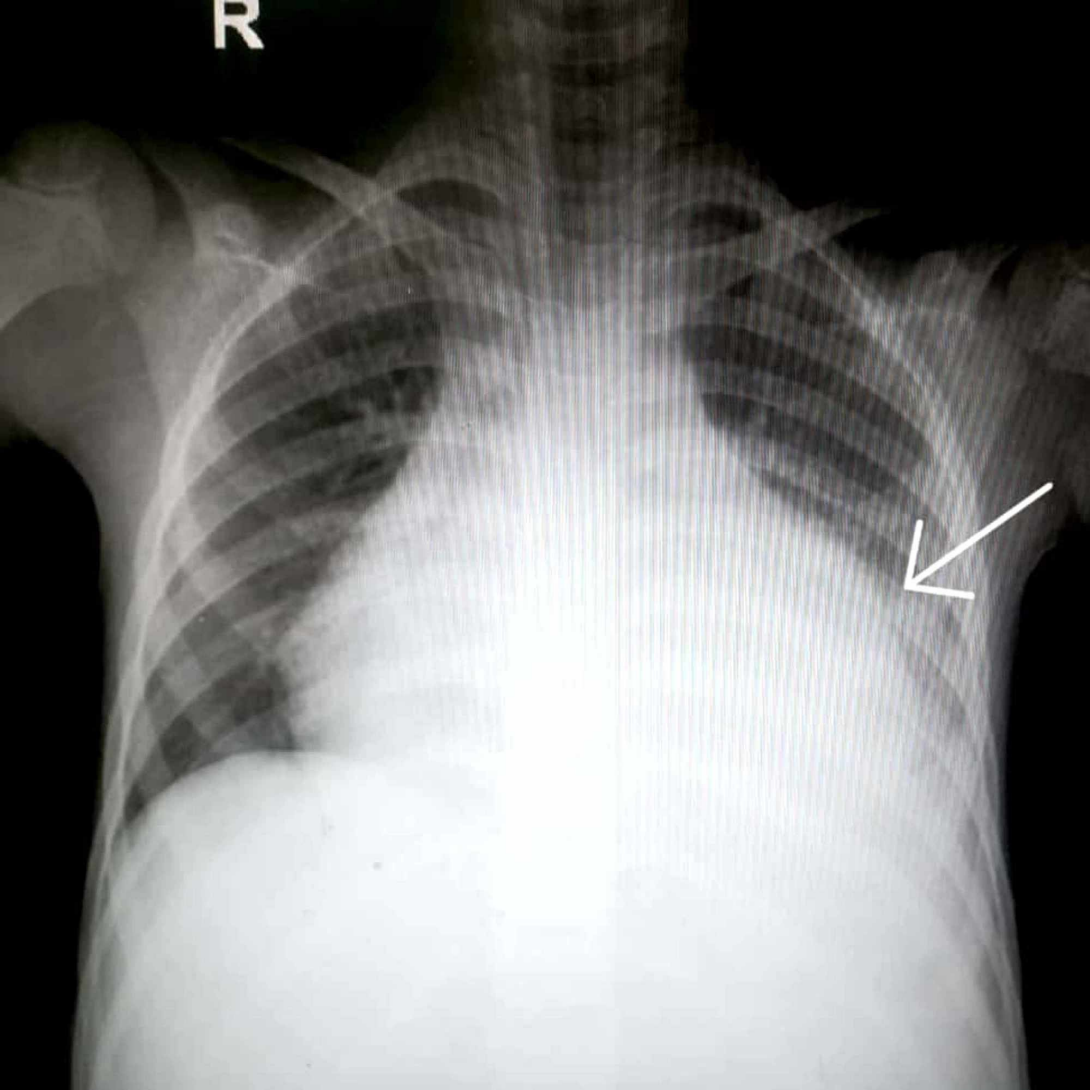
Chest X-ray showing enlargement of the cardiac silhouette with clear lung fields

A few days later, the child developed chest pain and respiratory distress. Cardiovascular examination revealed tachycardia, blood pressure of 106/70 mmHg, and bulging precordium. Auscultation revealed a prominent pericardial rub, no thrill, and normal heart sounds; the apex beat was difficult to localize. The chest was bilaterally clear to auscultation; there were no clinical signs of cardiac tamponade. Chest X-ray (CXR) showed enlargement of the cardiac silhouette with clear lung fields, as shown in Figure 2. Echocardiography showed mild to moderate pericardial effusion. Moreover, cardiac markers such as creatine kinase myocardial band (CK-MB) and Troponin I & T were within the normal range.

**Figure 3:**
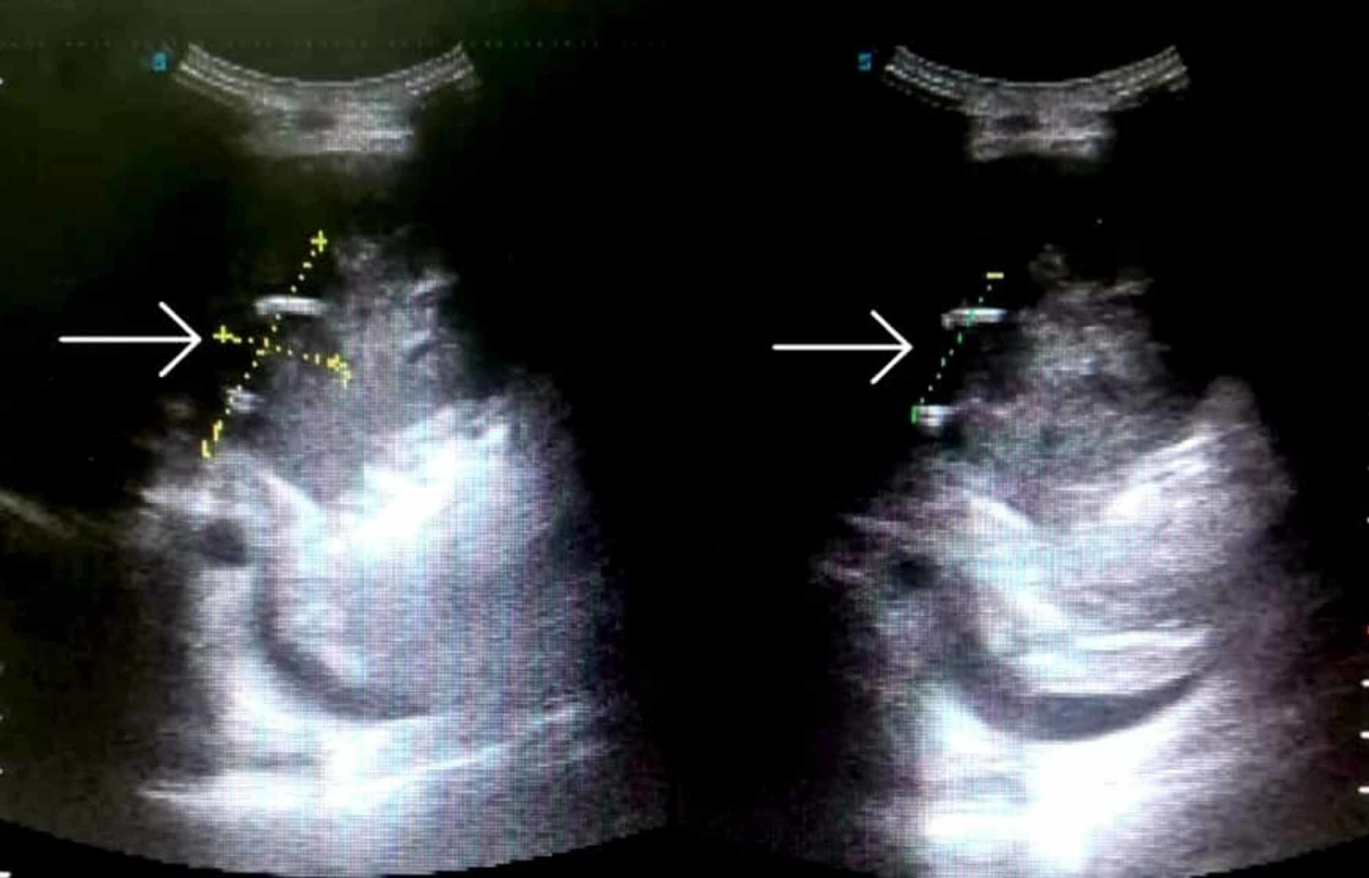
Abdominal ultrasound showing a residual abscess in the right lobe of the liver

Follow-up US revealed a residual abscess in the right lobe of the liver, measuring 4.4 x 2.2 x 2.9 cm, as shown in Figure 3. On consultation with the cardiologist, it was agreed upon that the effusion was mild and did not compromise cardiac function. The patient was given supportive care and managed conservatively with intravenous antibiotics and diuretics. Metronidazole was given for 14 days, followed by diloxanide furoate for the next seven days. The effusion gradually resolved following conservative treatment, and the patient was discharged in good condition. Subsequent follow-up at two weeks showed no signs of a residual liver abscess or pericardial effusion. 

## Discussion

ALA is the most common extra-intestinal manifestation of amoebiasis, which is an infection caused by the protozoan *E. histolytica*. ALA occurs when trophozoites of *E. histolytica* migrate from the intestine to the liver via the portal vein. The organism causes hepatic inflammation, followed by necrosis, which results in an abscess formation. It is rare in children relative to adults, typically occurring between the ages of 31 to 50 years, with a male to female ratio of 10:1. The major predisposing factors for ALA, especially in children, are malnutrition, unhygienic practices, overcrowding, and poor living conditions [1,2]. Our patient came from a low socioeconomic background, and the only apparent risk factor was a history of acute gastroenteritis hinting at unhygienic practices and poor sanitary conditions. Although the mortality rate of the liver abscess has decreased over time due to improved diagnostic and treatment modalities, associated complications such as rupture, sepsis, or dissemination may prove fatal. Pleuropulmonary complications are the most common, followed by intraperitoneal rupture of abscess in incidence. Pericardial and cerebral amoebiasis are relatively rarer complications [3,4].

Pericardial amoebiasis, although rare, is a grave complication with a high mortality rate. Incidence of pericarditis, as reported by Balasegaram, is as low as 1.5%, while that reported by Adam and Macleod from the largest experience of 2074 patients in South Africa is 1.3% [5,6]. Although the majority of ALAs are solitary in nature and involve the right lobe of the liver, amoebic abscesses of the left liver lobe are more likely to rupture into the pericardial space, which makes them an important clinical entity [7]. Thus, ALAs of the left lobe might prove to be life-threatening and, therefore, warrant early management with caution [3,4]. The presence of an abscess in the left lobe and associated pericardial effusion in our case was a rare entity; however, with timely diagnosis and treatment, we managed to avoid any fatal outcomes.

Lamont and Pooler have explained three stages in amoebic pericarditis [8]. In the first stage, there is a sympathetic pericardial effusion attributed to the inflammation caused by the abscess, which is usually in the left lobe of the liver. This stage demonstrates minimal pericardial effusion with exudative fluid, as seen in our case, and serves as a warning to the possible rupture of abscess into the pericardium. This first stage of pericarditis will resolve once the abscess is treated successfully. The second stage shows a suppurative pericardial effusion resulting from the rupture of the abscess into the pericardial space. The third stage is constrictive pericarditis, which develops over a period of weeks to months but has high mortality [2,8].

The clinical picture of ALA is highly variable and inconsistent. Children usually present with a subacute or acute history of abdominal pain along with fever and often chills. Moreover, patients may also have nausea, vomiting, cough, or dyspnea. On inspection, hepatomegaly with point tenderness is typical for liver abscesses. Jaundice is relatively uncommon, but its presence suggests a more severe course of the disease [2,3]. Diarrhea in children as a symptom of ALA is infrequent. A study reported that diarrhea occurred in only 20% of children with ALA [9]. Our patient presented with typical symptoms of ALA. He also had a three-day history of loose stools prior to hospital admission. Also, as mentioned earlier, our patient later developed chest pain and some dyspnea with prominent pericardial rub, hinting at pericardial effusion, which was further investigated and proven through radiographic evidence [4]. As evident from our case, laboratory investigations of patients with ALA typically show anemia, raised ESR, and leukocytosis in the absence of eosinophilia. Studies have also shown the presence of hypoalbuminemia. There may also be deranged liver enzymes, particularly alkaline phosphatase, directing towards the involvement of the liver [3,10,11]. Our patient, however, had normal liver enzyme levels. 

The confirmatory diagnosis of an amebic liver abscess is made through a combination of characteristic findings seen on imaging and serologic testing. Imaging modalities to assess a liver abscess include US, computed tomography (CT) scan, magnetic resonance imaging (MRI), and scintigraphy. The US shows a round, hypo-echoic mass; it may sometimes have heterogeneous echotexture. However, radiographic findings do not distinguish ALA from other causes of liver abscess; therefore, they should be followed by serological tests such as indirect fluorescent antibody and ELISA [11]. A positive ELISA test led us to our corroborative diagnosis of ALA. The presence of cysts of *E. histolytica* in stool further strengthened our diagnosis. Stool microscopy, although a helpful test, is an unreliable investigation as it is often negative for *E. histolytica* [12]. In addition, electrocardiography (ECG) findings and echocardiography may show evidence of pericardial effusion. US, CXR, and CT scan usually confirm liver abscess and fluid collection in the pericardial sac as increased echogenicity [4]. We performed CXR and echocardiography to evaluate our patient. The findings were consistent with pericardial effusion.

It may be reasonable to start treatment early in patients suspected of ALA. The drug of choice for ALA is metronidazole for a total duration of 10 days, which provides a cure rate of more than 90% [3]. It is usually supplemented with luminal amoebicides such as paromomycin, diloxanide furoate, or iodoquinol for intestinal disease. Aspiration of the liver abscess can be done either by percutaneous needle aspiration or by percutaneous catheter drainage. Studies have shown that percutaneous catheter drainage is superior to needle aspiration in providing success rates and quicker resolution [11,13]. In cases of pericardial effusion, early management of liver abscess usually resolves the inflammatory fluid in the pericardium [4]. Evident from this case, the patient was successfully managed conservatively with metronidazole and diloxanide furoate, as indicated by the resolution of effusion, thus, discharged in good condition.

## Conclusions

ALA of the left lobe of the liver bears a potential risk of extension into the pericardial cavity, causing effusion. It is a potentially life-threatening complication of ALA. Thus, although rare, pericardial amoebiasis should be highly suspected in patients with left lobe liver abscess developing cardiac symptoms. Sympathetic pericardial effusion is a mild initial stage of the pericardial spread of the disease. If the amoebic liver abscess is treated successfully at this stage, effusion will resolve eventually on its own without any need for pericardial fluid drainage. Therefore, knowledge of this condition, the need for prompt diagnosis, and timely management can save a valuable life.
